# Negative incidental emotions augment fairness sensitivity

**DOI:** 10.1038/srep24892

**Published:** 2016-04-22

**Authors:** Cuizhen Liu, Jing Wen Chai, Rongjun Yu

**Affiliations:** 1 School of Psychology and Center for Studies of Psychological Application, South China Normal University, China; 2 Department of Psychology, National University of Singapore, Singapore; 3 Neurobiology/Ageing Programme, Center for Life Sciences, National University of Singapore, Singapore; 4 Institute for Neurotechnology (SINAPSE), Center for Life Sciences, National University of Singapore, Singapore

## Abstract

Previous studies have shown that task-unrelated emotions induced incidentally exert carryover effects on individuals’ subsequent decisions in financial negotiations. However, the specificity of these emotion effects are not clear. In three experiments, we systematically investigated the role of seven transiently induced basic emotions (disgust, sadness, anger, fear, happiness, surprise and neutral) on rejection of unfair offers using the ultimatum game. We found that all negative emotions (disgust, sadness, anger and fear), but not happiness or surprise, significantly increased rejection rates, suggesting that the effect of incidental negative emotions on fairness is not specific to the type of negative emotion. Our findings highlight the role of fleeting emotions in biasing decision-making processes and suggest that all incidental negative emotions exert similar effects on fairness sensitivity, possibly by potentiating attention towards negative aspects of the situation.

When writing a paper, one may incidentally receive an unrelated phone call telling you of a good news, hear of a sad breaking news on the TV, or notice a man with an angry expression passing by. Although these events are incidentally and unrelated to our current goals and actions, they may still influence our emotions and behaviors^1^. Importantly, researchers have found that incidental emotions pervasively carry over from one situation to the next, affecting decisions that should be unrelated to that emotion, a process known as the carryover of incidental emotion^2^,^3^,^4^. For example, incidental fear modulates individuals’ preference for delayed reward, while incidental sadness and disgust influenced the endowment effect, even though such fleeting emotions should have no rational effect on the intertemporal choices or economic transactions of an individual^5^,^6^.

Yet, in real-life situations, many of our economic decisions are made within a social context. Emotions are inherently social, and a full explanation of the adaptive utility of transient emotions in influencing decisions requires an understanding of their reciprocal influence on interaction with partners^3^. Ironically, preference for fairness is often in conflict with self-interest, creating a social dilemma in which individuals have to choose either to be unfair by maximizing their own rewards or to be treated fairly. For example, in the ultimatum game^7^,^8^, the responder chooses to either accept or reject the reward allocation offered by a proposer. Upon acceptance, the resource is divided according to the offer. Rejection however leads to null reward for both players. Research with the ultimatum game shows that responders commonly accept offers of 50%, whereas lower offers are increasingly rejected as an act of social punishment^9^. Such rejection is significantly related to negative emotions (task-related integral emotion) induced by unfair offers, especially that of disgust and anger^10^,^11^.

The effects of incidental emotions on decision-making has been widely studied in many ways. Notably, early studies adopted the valence-based approach by classifying emotions into two broad categories–positive and negative^12^. Typically, these studies utilize a separate task of mood induction and participants are usually instructed that the mood induction task was irrelevant to the actual task. Importantly, these studies showed highly contrasting effects of mood valence on social perception. For instance, positive mood led to more optimistic judgements while negative mood led to more pessimistic judgements in risk perception^13^. Yet, the valence-based approach is often criticized for overgeneralizing the specific effects of emotions on cognition. For instance, it has been posited and validated that although two differing specific emotions of the same valence can produce different effects on decisions due to differing appraisals^14^. Focusing on the domain of social rejection, previous research of carryover effect of incidental emotions in the ultimatum game mainly focused on two specific negative emotions – sadness and disgust. Moretti and di Pellegrino^15^ demonstrated that relative to being in a sad or neutral mood, induced feelings of disgust significantly increased rejection rates of unfair offers and such effects were selectively related to interactions with a human partner rather than computer offer. Bonini, *et al.*^16^, however, found a lower rejection rate of unfair offers in the room with disgusting smell compared to the room with no particular smell. The main difference between the two studies lies in the methods used to induce transient emotion, one by using emotional images and the other by using unpleasant odor. It is worth noting that emotional visual and olfactory stimuli engage different discrete sensory-specific subregions, which may potentially produce different effects on subsequent decision-making^17^. Although Moretti and di Pellegrino did not a find significant effect of induced sadness on rejection rates, another two studies demonstrated that induction of incidental sadness by watching of video clips increased rejection rates towards unfair offers in the ultimatum game^18^,^19^. Only one study reported that induced anger produced higher rejection rates than induced happiness in a one-shot ultimatum game^20^. Thus, the role of specific negative emotion on fairness sensitivity remains unclear.

Since there are isolated studies showing that disgust, sadness, and anger increased rejection rates although using different methods of induction (Table 1), it raises the question whether all negative emotions have similar effects on fairness sensitivity. This is not an unwarranted assumption given that earlier studies have shown that two negative emotions increases rejection rates in the UG although clear inferences could not be drawn due to differing methodologies of emotion induction. If emotions indeed have a general effect on social rejections based on valence, it should be predicted that any emotions that carry the same valence would produce similar rejection rates in the UG. Concurring and advancing earlier studies, we critically hypothesize that only negative but not positive transient emotions would increase rejection rates and that there would be little difference when comparing across emotions of the same valence. Thus, the main purpose of our research is to systematically investigate how seven basic emotions (negative: sadness, disgust, anger, fear; positive: happiness, surprise; and neutral)^21^ modulate interpersonal economic decision-making in the ultimatum game using a common method of induction.

Notably, to our knowledge, previous studies to date have all adopted a between-subjects design to study the impact of incidental emotion on decision-making. That is, participants are first induced to immerse in a specific enduring mood by watching film clips, reading autobiographical stories, or even observing other’s feelings, and then conduct different cognitive tasks^18^,^22^,^23^. However, it is not clear whether transient displayed by emotional cues (i.e., emotional faces) could modulate decision processes during completion of cognitive tasks. Studies have revealed that when people are exposed to emotional facial expressions, they tend to mimic the facial stimuli, which subsequently leads to the assimilation of a congruent emotion^24^,^25^. If indeed the intuitive nature of emotional processing can differentially affect moral judgment of unfairness, it is reasonable to believe that subtle emotional cues from the environment may bias people’s final judgments. Thus, if emotional priming is effective, it is possible that corresponding emotional states play a mediating role in interpersonal economic decisions. In Experiment 1, we utilized such an affective priming task to examine whether and how incidental disgust and sadness influence responders’ decisions in the ultimatum game. In Experiment 2, using the similar paradigm, we extended our research to other important negative emotions: anger and fear. Finally in Experiment 3, we contrasted the findings of the two earlier experiments to a study of another two basic emotions of opposite valence: happiness and surprise.

## Experiment 1

### Method

#### Participants

Eighty-three (47 males) individuals participated in return for payment. Their ages ranged from 18 to 26 years with a mean of 19.61 (SD = 1.59). All participants were right-handed and reported no history of cognitive disorder or psychiatric illness. They all gave written, informed consent. The study was approved by the Ethics Committee of the School of Psychology at South China Normal University and was carried out in accordance with the approved guidelines.

#### Procedure

**Figure 1:**
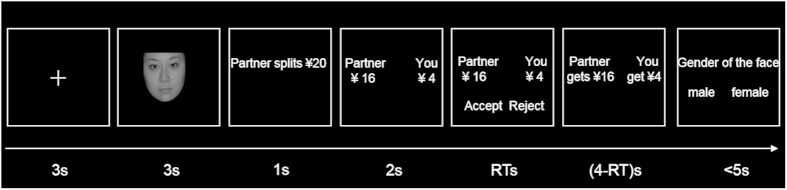
Experimental task design. First, an emotional face (varied between disgusted, sad and neutral emotions) was presented for 3 seconds. Then the proposer split a sum of money in 1 second and the given offer was displayed for 2 second. Participants determined to accept or reject this offer within 4 seconds followed by corresponding outcome feedback. A few seconds later, participants judged the gender of the face presented earlier. Note: Faces applied in our experiment were drawn from the Chinese Affective Face Picture System (CAFPS). However, the neutral face in this figure are not from the CAFPS, but is comparable to the ones used in the task. Cuizhen Liu took and processed the face image of Shanshan Liu, who gave written consent for publication in Scientific Reports.

A 3 (fairness: fair/moderately unfair/extremely unfair) × 3 (emotional priming: disgust/sadness/neutral) within-subject factorial design was employed. Participants performed the ultimatum game that is intermixed with different emotional primers (Fig. 1 for schematic illustration of trial design). At the start of the experiment, participants were informed that they would participate in two intermixed tasks: the ultimatum game and the facial gender recognition task. Each participant was made aware of the rules of the ultimatum game. All participants played the role of responder who chose either to accept or reject the reward allocation offer proposed by a proposer. Accepting the offer led to the division of the money according to the offer, whereas choosing rejection resulted in both players receiving nothing. Participants were paid the basic payment for participation plus a fixed proportion of money selected randomly from one trial. For the facial gender recognition task, participants were asked to perform a judgement of gender for each face, which was intercrossed in the ultimatum game. At the end of the ultimatum game, accuracies on the identification of gender would be presented immediately, therefore participants had to view each faces earnestly in order to guarantee a high accuracy. It is important to note that this gender identification task was designed to ensure that participants hold the target expression in memory, with the goal of increasing the impact of the emotional priming. The participants’ accuracy also served to verify their engagement in the priming task. After that, participants completed an emotional manipulation check task.

#### Emotional priming

Each trial started with the presentation of a fixation cross in the center of the screen for 3 seconds (Fig. 1). After which, the cross was replaced with a picture of a face for 3 seconds. The face materials were selected from the Chinese Affective Face Picture System (CAFPS), a standardized and validated set of Chinese facial expression photographs which has been widely applied in studies involving Chinese participants^26^. All pictures were in black-and-white and were taken with models looking straight ahead with different types of expressions (neutral and six basic facial emotions: disgust/sadness/fear/anger happiness/surprise). Participants rated the intensity of the faces from 1 to 9 (1 = low intensity, 9 = high intensity). CAFPS was used in order to avoid the effect of ethnicity (people are better at recognizing faces from their own ethnic background relative to faces of other ethnicity). Fifty-four emotional face photos, each 18 portraying neutral, sad and disgusted emotions respectively, were selected from CAFPS. The average intensity score of selected neutral face photos was 5.80 (SD = 0.21), sad face was 6.00 (SD = 0.64) and disgusted face was 6.55 (SD = 0.55). Both genders were equally represented in each of the selected three categories of facial expressions. Each picture was presented with a picture size of 260 × 300 pixels at a viewing distance of 60 cm. Participants were instructed to carefully observe the faces as they would have to perform a gender recognition. In actuality, viewing the images served as transient emotional priming.

#### The ultimatum game

After the emotional priming, participants then played the ultimatum game as the role of responder, receiving one-time monetary offers from 54 different proposers, presented in a pseudorandomized order. The distribution offer was displayed for 2 seconds, with the names of the proposer and responder displayed to increase the verisimilitude for participation. Participants then decided to accept or reject this distribution offer by pressing the corresponding response buttons within 4 seconds. Their outcomes were then displayed indicating how much money they had gained (e.g., if accepting, the result will be ‘you get ¥4, your partner get ¥16’; if rejection, ‘you get ¥0, your partner get ¥0.’). We told participants these offers were collected from participants in a previous experiment and that there were different proposers for each trial of the game. Each trial was therefore treated to be a “one-shot” game. Research of the ultimatum game has shown that telling participants the offers were made by other study participants is an effective manipulation^19^. The sum of money ranged from ¥10 to ¥27 (about 1.6~4.4 US dollars). Unknown to the participants however, the division proposals were manipulated by the experimenter with 18 fair allocations (about 50% to responder), 18 moderately unfair allocations (about 35% to responder), and 18 extremely unfair offers (about 15% to responder). Therefore, each emotional priming condition (disgust, sadness, neutral) includes 18 trails respectively, 6 fair offers (the amount to proposer vs responder was ¥6: 6, 8: 9, 10:10, 11: 10, 12:12, 13:13), 6 moderately unfair offers (¥7:4, 9:5, 10:6, 12:6, 13:6, 17:10), and 6 extremely unfair offers (¥9:1, 11:2, 12:3, 19:3, 20:3, 20:5). Participants then judged the face’s gender after each round of the ultimatum game.

#### Emotional manipulation check

To assess whether face expressions presented in the procedure are effectively perceived by their corresponding emotions, participants conducted an emotional rating task to these faces after the experiment. They were asked to report the intensity of each facial emotion along a 9-point scale from1 to 9 (1 = low intensity, 9 = high intensity) on 5 different emotions including disgust, sadness, anger, fear and neutral.

After the experiment, participants completed the Behavioral Activation System (BAS)/ Behavioral Inhibition System (BIS) scale, a questionnaire that assesses individual variability in trait approach and withdrawal motivation^27^. Subsequently, a close debriefing revealed that no participant was aware of the actual purpose of the study. Participants also expressed no suspicion regarding the offer distributions in the ultimatum game. After completion of the total procedures, each participant received a base payment of ¥10 plus the sum of the extra reward from the randomly chosen trial.

## Results

### Priming effect in the ultimatum game

Mean rejection rate was adopted to calculate participants’ responses for each condition. We conducted a 2-way repeated-measures ANOVA on rejection rates with emotional primes (disgust/sadness/neutral) and fairness (fair/moderately unfair/extremely unfair) as within-subject factors. The main effect of fairness [F(2, 164) = 186.58, p < 0.001, η_p_^2^ = 0.70] and the main effect of emotional priming [F(2, 164) = 4.19, p = 0.017, η_p_^2^ = 0.05] were significant. Not surprisingly, participants rejected more distributions for extremely unfair trials (mean ± SE, 0.70 ± 0.04) than for moderately unfair trials (mean ± SE, 0.30 ± 0.03), p < 0.001, and rejected more for moderately unfair trials than for fair trials (mean ± SE, 0.03 ± 0.01), p < 0.001. Participants rejected significantly greater in disgust condition (mean ± SE, 0.36 ± 0.02) than in neutral condition (mean ± SE, 0.33 ± 0.02), p = 0.003, and the rejection rates were not reached significant between sadness condition (mean ± SE, 0.35 ± 0.02) and neutral condition, p = 0.084, as well as between sadness condition and disgust condition, p = 0.281. Moreover, there was no interaction between fairness and priming [F(4, 328) = 1.58, p = 0.180, η_p_^2^ = 0.02].

**Figure 2:**
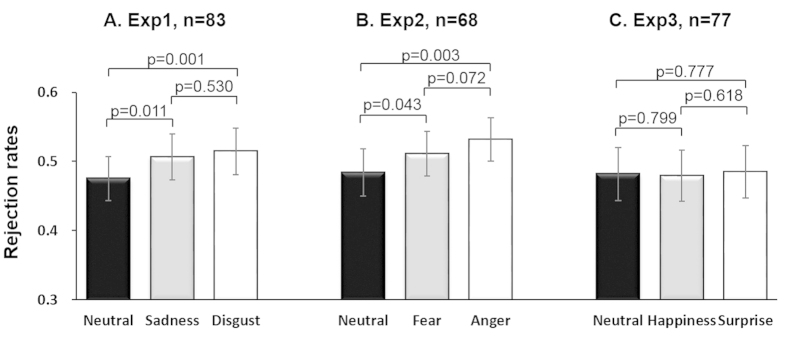
Rejection rates of unfair offers (after emerging two unfair levels) in three priming conditions for each experiment.

For further analysis, we merged the two unfair conditions and performed a 2 (fair/unfair) × 3 (disgust/sadness/neutral) repeated-measures ANOVA. We also found a main effect of fairness [F(1, 82) = 208.09, p < 0.001, η_p_^2^ = 0.72] and a main effect of priming [F(2, 164) = 3.16, p = 0.045, η_p_^2^ = 0.04]. Importantly, there was a significant interaction between fairness and priming [F(2, 164) = 4.52, p = 0.012, η_p_^2^ = 0.05]. Pairwise comparisons revealed that for unfair offers, participants rejected more when previous faces were of disgust (mean ± SE, 0.52 ± 0.03) than when they were neutral (mean ± SE, 0.48 ± 0.03), p = 0.001; they also rejected more unfair offers in sadness condition (mean ± SE, 0.51 ± 0.03) than in neutral condition, p = 0.011 (Fig. 2A); there was no significant difference between sadness condition and disgust condition, p = 0.530. In addition, we conducted a similar ANOVA for reaction time and found no effects on emotional factors (p values > 0.1). The priming effects of gender on rejection rate were also not significant (p values > 0.1).

### Manipulation check

**Figure 3:**
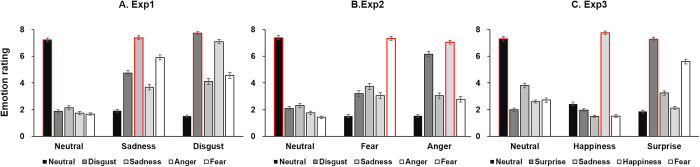
Mean intensity scores for three kind of priming faces on five emotions in each experiment.

The intensity ratings of the selected three target emotional faces on five emotions demonstrated the effectiveness of our emotional manipulation (Fig. 3A). As expected, participants rated disgusted faces as more disgusting (M = 7.73, SD = 0.97) than sad (M = 4.11, SD = 1.98), t_82_ = 16.79, p < 0.001. Similarly, the sad expressions were reported to be more sad (M = 7.38, SD = 1.35) than disgusted (M = 4.74, SD = 1.87), t_82_ = 11.04, p < 0.001. For neutral faces, participants also reported more neutral (M = 7.22, SD = 1.20) than both disgust (M = 1.86, SD = 1.07), t_82_ = 27.61, p < 0.001, and sadness (M = 2.14, SD = 1.40), t_82_ = 21.79, p < 0.001. Participants’ mean accuracy on gender identification was 88% (SD = 0.07). This was significantly higher than random (p < 0.001), indicating that participants were sufficiently engaged in the emotional priming task. Results of the questionnaire also revealed no statistical correlation between BAS/BIS scores and rejection rates on the three affective priming conditions (p values > 0.1), thus confirming that rejection differences across emotional conditions cannot be attributed to participants’ preexisting behavioral trait differences.

## Experiment 2

Previous studies reported on the effects of specific transient emotions on fairness perception. Experiment 1 confirmed that indeed, negative emotions of disgust and sadness led to higher rejection rates in the UG. However, it remains to be answered whether these effects were emotion specific, or that negative emotions in general have a common effect on rejections. If the alternative hypothesis is true, we predict that other emotions of negativity would also produce similar rejection rates in the UG. Therefore, in Experiment 2 we proceeded to investigate the effects of another two basic negative emotions, anger and fear.

### Participants and Procedure

Sixty-eight healthy participants (mean age ± SD, 20.40 ± 1.64 years) participated. They all gave written, informed consent. The study was approved by the Ethics Committee of the School of Psychology at South China Normal University and was carried out in accordance with the approved guidelines. The procedure was the same as that reported in Experiments 1 except that disgusted and sad faces were replaced with angry and fearful faces that were also selected from the CAFPS. A 3 (fairness: fair/moderately unfair/extremely unfair) × 3 (emotional priming: fear/anger/neutral) within-subject factorial design was employed. The average intensity score of selected angry faces was 6.75 (SD = 1.02) and fearful face was 6.63 (SD = 0.72).

## Results

### Priming effect in the ultimatum game

Once again, the rejection rates obtained in the UG was submitted to a 3 (fairness) X 3 (emotional priming) ANOVA for repeated measures. A main effect of fairness [F(2, 134) = 226.09, p < 0.001, η_p_^2^ = 0.77] and a main effect of emotional priming [F(2, 134) = 6.34, p = 0.002, η_p_^2^ = 0.09] were found. Participants rejected more distributions for extremely unfair trials (mean ± SE, 0.77 ± 0.04) than for moderately unfair trials (mean ± SE, 0.25 ± 0.04), p < 0.001, and rejected more for moderately unfair trials than for fair trials (mean ± SE, 0.01 ± 0.004), p < 0.001. Participants rejected greater in fearful priming condition (mean ± SE, 0.35 ± 0.02) than in neutral condition (mean ± SE, 0.33 ± 0.02), p = 0.026, and they also rejected more in angry priming trials (mean ± SE, 0.36 ± 0.02) than in neural trials, p = 0.003; no significant difference was found between fear condition and anger condition, p = 0.133. There was no interaction between fairness and priming [F(4, 268) = 1.69, p = 0.152, η_p_^2^ = 0.03].

Merging the two unfair conditions, a 2 (fair/unfair) × 3 (fear/anger/neutral) repeated-measures ANOVA also revealed a main effect of fairness [F(1, 67) = 270.10, p < 0.001, η_p_^2^ = 0.80] and a main effect of priming [F(2, 134) = 5.59, p = 0.005, η_p_^2^ = 0.08]. However, there was a significant interaction between fairness and priming [F(2, 134) = 4.37, p = 0.014, η_p_^2^ = 0.06]. Pairwise comparisons revealed that participants rejected more unfair offers when previous faces were fearful (mean ± SE, 0.51 ± 0.03) than when they were neutral (mean±SE, 0.48 ± 0.03), p = 0.043; They also rejected more unfair offers in anger condition (mean ± SE, 0.53 ± 0.03) than in neutral condition, p = 0.003; there was a slightly difference between fear priming and anger priming conditions in unfair trials, p = 0.072 (Fig. 2B). No priming effect was found on reaction time and gender (p values > 0.1). Taken together, results in Experiment 2 involving negative emotions of anger and fear also produced a similar pattern of results as in Experiment 1 which involved disgust and sadness. This provided critical support that negative emotions have a general effect on fairness perception.

### Manipulation check

To assess the effectiveness of emotional manipulation, participants rated fearful faces (M = 7.33, SD = 1.70) as more fearful than angry (M = 3.06, SD = 1.87), t_67_ = 18.20, p < 0.001, and rated angry faces as more angry (M = 7.05, SD = 1.18) than fearful (M = 2.77, SD = 1.66), t_67_ = 18.64, p < 0.001, thus confirming the effectiveness of the emotion manipulation (Fig. 3B). Participants’ mean accuracy on gender identification (89%, SD = 0.06) was significantly higher than random (p < 0.001), indicating that participants were sufficiently engaged in the emotional priming task. No statistical correlation between BAS/BIS scores and rejection rates on the three affective priming conditions (p values > 0.1) was obtained, indicating that participants’ preexisting behavioral trait differences did not contribute to rejection differences across emotional conditions.

## Experiment 3

Results from Experiment 1 and 2 showed that all four basic negative emotions exerted similar effects on decision-making in the ultimatum game. This raise the question whether all high arousal emotions can influence fairness concern in a similar way regardless of its valence (positive or negative). Thus, in Experiment 3, we further investigated the role of happiness and surprise in the ultimatum game.

### Participants and Procedure

Seventy-seven healthy participants (mean age ± SD, 20.53 ± 2.08 years) participated. They all gave written, informed consent. The study was approved by the Ethics Committee of the School of Psychology at South China Normal University and was carried out in accordance with the approved guidelines. We employed a 3 (fairness: fair/moderately unfair/extremely unfair) × 3 (emotional priming: happiness/surprise/neutral) within-subject factorial design, and the procure details were same with Experiment 1 and 2. The average intensity score of selected happy faces from CAFPS was 6.59 (SD = 0.50) and the surprise faces was 5.45 (SD = 0.72). After the ultimatum game, participants performed the emotional rating task to these faces on five different emotions including surprise, sadness, happiness, fear and neutral.

## Results

### Priming effect in the ultimatum game

Repeated-measures ANOVA on rejection rates with emotional primes (happiness/surprise/neutral) and fairness (fair/moderately unfair/extremely unfair) as within subjects factors revealed a main effect of fairness [F(2, 152) = 135.83, p < 0.001, η_p_^2^ = 0.64]. Once again, participants rejected more distributions for extremely unfair trials (mean ± SE, 0.68 ± 0.04) than for moderately unfair trials (mean ± SE, 0.28 ± 0.04), p < 0.001, and rejected more for moderately unfair trials than for fair trials (mean ± SE, 0.03 ± 0.01), p < 0.001. However, the main effect of emotional priming [F(2, 152) = 0.18, p = 0.839, η_p_^2^ = 0.002] and the interaction between fairness and priming [F(4, 304) = 0.89, p = 0.468, η_p_^2^ = 0.012] were not significant, suggesting that transient emotions of positivity do not affect perceptions of fairness.

To further confirm this observation and to be consistent with Experiments 1 and 2, we proceeded to merge the two unfair conditions and performed a 2 (fair/unfair) × 3 (happiness/surprise/neutral) repeated-measures ANOVA. This also revealed a main effect of fairness [F(1, 76) = 172.22, p < 0.001, η_p_^2^ = 0.69]. Unlike previous experiments involving negative emotions, however, the main effect of priming [F(2, 152) = 0.35, p = 0.706, η_p_^2^ = 0.01] and the interaction between fairness and priming [F(2, 152) = 0.08, p = 0.927, η_p_^2^ = 0.001] were not reached significant (Fig. 2C). No priming effect was found on reaction time and gender (p values > 0.1). This confirmed that only transient emotions of negative valence, but not positive or mixed emotion affected peoples’ perception of fairness.

### Manipulation check

Participants rated happy faces (M = 7.76, SD = 1.02) as more happy than surprising (M = 1.97, SD = 1.18), t_76_ = 29.19, p < 0.001, and rated surprise faces as more surprising (M = 7.28, SD = 1.20) than happy (M = 2.13, SD = 0.98), t_76_ = 31.42, p < 0.001, thus confirming the effectiveness of the emotion manipulation (Fig. 3C). The mean accuracy on gender identification (92%, SD = 0.05) was significantly higher than random (p < 0.001). There was no statistical correlation between BAS/BIS scores and rejection rates on the three affective priming conditions (p values > 0.1).

## Discussion

It is undeniable that people are often bombarded by incidental emotions that are not relevant to their current goals when making decisions. It is therefore essential to understand how people’s transient emotional states influence the way they make decisions. In three experiments, compared with the neutral condition, we found that induced negative basic emotions (i.e., disgust, sadness, anger and fear) all increased the rejection rate of unfair offers in a similar pattern, whereas induced happiness and surprise had no effect compared with the neutral condition. Our results therefore suggest that negative emotions generally augment individuals’ sensitivity to fairness and challenge previous findings that disgust or sad selectively modulates fairness sensitivity in the ultimatum game^3^,^6^.

Our findings challenge the approach-withdrawal motivational theory which proposes that organisms’ emotional system can be subdivided into an aversive and appetitive apparatus (respectively promoting defensive and approach behavior)^28^,^29^. It is worth noting that disgust, sadness and fear promotes a withdrawal tendency whereas anger promotes an approach tendency^30^,^31^. However, the fact that they all lead to similar decision patterns regarding unfair offers lends credence to the hypothesis that a negative valence construct, and not just approach/withdrawal motivation, plays a role in biasing decisions in the ultimatum game. It is thus not surprising that incidental disgust could strengthen the decision’s emotion induced by the unfairness offers, and drive a higher rejection rate. Anger, associated with the desire to change the situation and move against another person, triggers aggression and provides the motivation to respond to injustice^32^, thus resulting in a high rejection rate of unfair offers^31^,^33^.

Most theories of affective influences on judgement and choice take a valence-based approach, contrasting the effects of positive versus negative feeling states^34^,^35^,^36^. Positive affect has been associated with high expectations about success and more optimistic framing, whereas emotions with a negative valence are usually associated with lower confidence and with a focus on negative rather than positive consequences^37^,^38^. Previous research has suggested that individuals in a negative mood are more negative and critical in their attitudes and judgment toward others and self^39^,^40^, even though the emotions have completely no relationship with decision tasks^13^. In the ultimatum game, the preference for fairness is often in conflict with self-interest, creating a social dilemma in which individuals have to choose either to maximize their own rewards or to be treated fairly. Accepting or rejecting an unfair offer is a complex decision that involve a significant degree of generative elaboration of the stimulus details such that decisions are likely to be infused by emotions^35^. Our findings are consistent with this hypothesis, namely that participants may focus their attention on the negative emotional consequences of unfair offers (i.e., being treated unfairly) rather than the positive impact of accepting such offers (i.e., monetary reward) in negative priming conditions, thereby disgust, sadness, anger and fear, all prompting higher rejection rates of unfair offers. Sadness is often experienced after pain and irrevocable losses. Thus, sadness may induce more careful and systematic processing that reflect a motivation to enhance the processing of information related to potentially threatening and harmful situations^18^,^39^. The main function of fear and anger is to act as a signal of impending threat and to trigger appropriate adaptive responses. Disgust, originating as a rejection response that protect the body from contaminating object, signals warning of both dangerous foods and immorality^6^,^30^. Thus, it is possible that all these fleeting negative emotions prime participants to pay more attention to the negative aspects of the unfair offers and change how negative information is weighted in the evaluation process. This is also consistent with our recent findings that individuals felt more dissatisfied about unfair (as opposed to fair) offers when fairness was emphasized using salient visual cues (unpublished data). But emphasizing the utilitarian aspect (e.g. winning monetary reward) of offers did not influence evaluations compared with the no emphasis condition. Thus, it is possible that the fairness dimension of offers is more malleable and thus is more sensitive to negative emotion priming.

There are several limitations of the present research. One concern is that whether the stimuli we used can successfully elicit the targeted emotion or not. Faces identified as belonging to one kind of emotion may also arouse other emotions, thus may confound the findings. For example, for disgust faces in Experiment 1, disgust ratings are very close to the anger ratings, that means participants also perceived disgust faces as depicting anger to some degree. Similarly, in Experiment 2, angry faces were also perceived as disgusted to some extent. However, the ratings between these ratings reached significant. The significant differences between emotional ratings at least guaranteed that faces we used mainly induced the targeted emotion on average, confirming the strong and discrete mood induction effects. Another concern is that participants may confuse the faces used to elicit emotions with the persons who made offers. We have explicated participants that those faces were selected from a database and were definitely unrelated to the ultimatum game in any sense. Post-experiment debriefing also confirmed that all participants understood and believed that those faces are not proposers’ faces. Although we cannot rule out the possibility that participants still unconsciously took those faces as proposers’ faces, we believe that such unconscious confusion is one mechanism that how incidental emotion works^12^. Thus, the observation that rejection rates in the UG were affected by transient negative emotions even though participants were consciously made aware that these faces were to be treated as task-irrelevant indicates the importance of taking into account transient emotional states in biasing decisions, whether or not this confusion was consciously perceived. In our study, participants were asked to identify the gender of human faces. This kind of task would likely suppress the emotional influence of faces, compared to those focusing on the emotional aspect of stimuli (e.g. emotion recognition task). Nevertheless, we still found robust effects of negative emotions on fairness sensitivity. Future studies may further investigate whether asking participants to pay attention to the emotional aspect of stimuli would produce stronger priming effects. Finally, the underlying mechanisms of the negative emotions on fairness need further exploration. A neuroimaging study revealed that receiving unfair offers while in a sad mood elicited activity in anterior insula, a brain area related to aversive emotional states and somatosensory integration^19^. Given our findings, insular activation uniquely might be the common currency mediating the general effect of negative emotions on social rejection^41^. It would be interesting if other negative emotions besides sadness as observed in Harle *et al.*^19^ also activate a common set of brain regions, especially the anterior insula when engaging in tasks of similar nature. Moreover, the current study did not examine the impact of positive and negative emotions on UG performance in the same experiment due to the duration of experiments. Future research may use within-subject design to compare the effects of emotions on fairness sensitivity.

Emotions often persist beyond the eliciting situation and affect subsequent behavior and cognition. To date, the psychological field of emotion science is undergoing a revolutionary phase that has already begun to impact theories of decision-making^3^. Our study suggests that the role of incidental negative emotions, though transient appeared, no matter what is consistent with the background moods of unfair offers in the ultimatum game, no matter what is approach-motivated or withdrawal-motivated, all have similar behavioral patterns in biasing decisions in the ultimatum game. Thus, global negative feelings intensify fairness sensitivity. Our study highlights that subtle emotions can permeate in interactive decision-making and the specific mechanisms driving such effects may be more complicated than previously thought and thus awaits further research.

## Additional Information

**How to cite this article**: Liu, C. *et al.* Negative incidental emotions augment fairness sensitivity. *Sci. Rep.* **6**, 24892; doi: 10.1038/srep24892 (2016).

## Figures and Tables

**Table 1:** The main findings of previous studies and the current study.

	Disgust	Sadness	Anger	Fear	Happiness	Surprise	Neutral
Harle & Sanfey^18^		↑			−		−
Andrade & Ariely^20^			↑ (vs. happy)				
Moretti & Pellegrino^15^	↑	−					−
Bonini *et al.*^16^	↓						−
Harlé *et al.*^19^		↑			−		−
The present study	↑	↑	↑	↑	−	−	−
